# Pulse Pressure: A Predictor of Intervention in Blunt Abdominal Trauma

**DOI:** 10.7759/cureus.41305

**Published:** 2023-07-03

**Authors:** Sumbla Salman, Osama Laeeque, Bushra Jawaid, Omer B Khalid, Hassan Shahab, Komal Faheem

**Affiliations:** 1 General Surgery, Dow University of Health Sciences, Civil Hospital Karachi, Karachi, PAK; 2 Orthopaedics and Traumatology, Liaquat National Hospital, Karachi, PAK; 3 Surgery, Dow University of Health Sciences, Civil Hospital Karachi, Karachi, PAK

**Keywords:** torso trauma, isolated blunt torso trauma, operative intervention, massive transfusion, low pulse pressure

## Abstract

Background: Patients with life-threatening hemorrhages due to blunt torso trauma are at a particularly high risk of being underdiagnosed. The pulse pressure (PP) starts narrowing down before the traditional parameters start changing, making it a useful tool for assessing and planning early intervention.

Objective: To assess the utility of low PP in predicting massive transfusion (MT) or operative intervention in patients with isolated blunt abdominal trauma.

Material and methods: A total of 186 patients were included. The PP and mean arterial pressure (MAP) were calculated. Vitals, PP, and MAP were monitored every 15 min during the first 6 h, then every 30 min during the next 6 h, and afterward, every 4 h until discharge. A Chi-square test and an independent t-test (as appropriate) were applied to compare variables with PP at the time of presentation. Differences were considered statistically significant at p-value ≤ 0.05.

Results: A total of 55.9% of these patients had injuries due to road traffic accidents (RTA). Emergency operative intervention was provided to 26.3% of the patients. Death was 4.3%. MT was required by 26.3% of the patients. There was a statistically significant association between low PP and sex, length of stay, repeat extended focused assessment with sonography in trauma (eFAST), emergency operational intervention, outcome, MT, number of crystalloids consumed within the first four hours after presentation, injury severity score, systolic blood pressure (SBP), and pulse rate.

Conclusion: The PP <30 mmHg was observed as a useful predictor for increased blood loss requiring blood transfusion or operative intervention.

## Introduction

Hemorrhage is the most common cause of death in injured patients [1]. Approximately 50-60% of the deaths caused by trauma occur within the first 24h of hospitalization [2]. Worldwide, approximately 1.9 million deaths per year are caused by hemorrhagic shock, of which 1.5 million deaths result from trauma [3].

However, the diagnosis of life-threatening bleeding may go unrecognized or there may be a delay in recognition, particularly among patients with blunt torso trauma [4]. Rapid identification of those who are bleeding is critical so the bleeding can be stopped and lost volume replaced. While external bleeding can easily be seen, identifying internal cavitary bleeding can be challenging [5].

As determined by low systolic blood pressure (SBP), hypotension is a well-documented indicator of ongoing blood loss requiring emergent therapeutic intervention [5]. Inconspicuous hemorrhage may be overlooked by medical staff. Without intervention, patients can develop sequential organ failure, coagulation dysfunction, and even death due to insufficient blood transfusion and fluid resuscitation in a short time [6]. Several physiologic variables have been evaluated and used to both identify and estimate the severity of blood loss [7]. In the setting of trauma, hypotension hastens clinical decision-making and yields early interventions such as blood transfusion [8] and operative intervention [9].

If medical staff can identify the condition of traumatic massive hemorrhage early, intervene quickly, and actively adjust the treatment strategy, the disability and mortality rate due to massive traumatic hemorrhage may be reduced, and outcomes of severe trauma may be improved [10]. Blood transfusions are frequently administered during the resuscitation of severely wounded combat casualties, especially within the first 6 to 12 hours, when death from hemorrhage is most likely to occur [11-14]. The classic definition of massive transfusion (MT) was defined during the Vietnam War as the transfusion of 10 units of blood in 24 hours. Timely activation of MT protocols is imperative during resuscitation [15].

Pulse pressure (PP), specifically a narrowed PP (defined as systolic arterial pressure minus diastolic arterial pressure <30 mmHg), may also indicate the presence of shock [3]. Previous research has demonstrated that pressure-derived stroke volume (SV) estimates such as PP may be a better indicator of hypovolemia than more traditional vital signs such as heart rate, blood pressure, and mean arterial pressure (MAP) [16,17]. Although PP has been used for determining fluid responsiveness [18], or as a predictor of cardiovascular disease [19,20] in the non-trauma setting, a gap currently exists in the literature regarding its utility as an early detector of hemorrhage in a normotensive trauma patient.

PP is defined as the difference between diastolic and SBP [21]. The difference between the two values can either become wider or narrower. In a bleeding patient, PP narrows as an early response to decreased intravascular volume. As the venous capacitance decreases, the measured diastolic blood pressure increases, before any change to the SBP is seen. What is considered to be a narrowed PP can also vary depending on age; older patients tend to have wider PPs due to a combination of decreased diastolic pressure and increased systolic pressure secondary to the stiffness of large arteries [21].

At present, limited research has been done on the clinical relevance of narrow PP in the trauma population to recognize the utility of narrowed PP in identifying trauma patients in hemorrhagic shock and predicting the need for MT. Utilizing our experience at our institute with a high incidence of isolated blunt torso trauma patients, we focused on studying the utilization of PP as an independent predictor of MT or operative intervention.

Pre-print

This article was previously posted to the Research Square preprint server on January 02, 2023.

## Materials and methods

In total, 186 patients were included in the study. This prospective observational study was conducted in a level 1 Trauma Center, from May 2022 to October 2022. Approval from the institutional ethical review committee has been secured. Before enrolment in the study, participants were briefed about the purpose of the research and its benefits. Written consent was taken. Patient’s medical record numbers were coded into serial numbers to conceal their identities. Patients of both genders aged at least 18 years had blunt abdominal trauma but were hemodynamically stable at the time of presentation in the emergency department (ED), were received and managed by the emergency department team and the trauma team was notified. The patient’s basic clinical assessment was carried out. Examination findings, blood pressure readings, pulse rate, Glasgow Coma Scale (GCS), RR, and oxygen saturation were recorded on a predesigned proforma. Afterward, PP and MAP were calculated and noted on the same proforma.

All appropriate hematological (such as complete blood count and arterial blood gases) and radiological investigations such as X-rays, ultrasounds, and computed tomography (CT scans) were effectuated to ascertain the extent and sites of injury. Narrow PP was defined as PP<30. However, MT was considered if any of the five conditions were fulfilled: (a) Replacement of one entire blood volume within 24 h, (b) Transfusion of >10 units of packed red blood cells (PRBCs) in 24 h, (c) Transfusion of >20 units of PRBCs in 24 h, (d) Transfusion of >4 units of PRBCs in 1 h when on-going need is foreseeable, or (e) Replacement of 50% of total blood volume (TBV) within 3 h. Patients’ management was followed during the entire length of their hospital stay and they were followed until discharge or demise. During their entire admission period, their vitals, pulse, and MAP were monitored every 15 minutes during the first 6 hours, then every 30 minutes during the next 6 hours. Afterward, vitals were monitored every 4 hours until discharge.

Data was entered in Statistical Product and Service Solutions (SPSS) (IBM SPSS Statistics for Windows, Version 21.0, Armonk, NY). Qualitative variables were presented as frequencies and percentages. Mean ± standard deviations were calculated to summarize normally distributed quantitative variables whereas non-normal numerical variables were summarized as medians with inter-quartile range. The assumption of normality was tested with the Shapiro-Wilk test. The Chi-square test was applied to compare categorical variables among patients with normal PP compared to those with low PP at the time of presentation. An independent t-test was applied to compare quantitative variables among the two study groups. A P-value less than or equal to a 5% level of significance was considered statistically significant.

## Results

A total of 186 patients were enrolled in the study, with a gender split of 165 (88.7%) men and 21 (11.3%) women. Most patients (77.4%) were under the age of 35. Most of the patients stayed ≤24 hours. It was observed that 55.9% of patients were injured due to road traffic accidents (RTA) followed by falls (32.3%) and assaults (11.8%). Co-morbid were not present among most (87.1%) of the patients. Among 85.8% of the patients, the results of eFAST were negative; on repeated eFAST done every 6 hours for the first 24 hours, the positive result increased to 44.6%. Emergency operative intervention was provided to 26.3% of patients. Death was observed among only 4.3% of patients. An MT was required by 26.3% of patients. The results are presented in Table 1.

**Table 1:** Frequency distribution of gender, age, clinical findings, emergency intervention, and transfusion

	Frequency n	Percentage (%)
Gender		
Male	165	(88.7)
Female	21	(11.3)
Age Group		
≤35 years	144	(77.4)
36-50 years	25	(13.4)
>50 years	17	(9.1)
Length of Stay		
≤24 hours	100	(53.8)
25-72 hours	43	(23.1)
>72 hours	43	(23.1)
Mechanism of injury		
Assault	22	(11.8)
Fall	60	(32.3)
Road Traffic Accident	104	(55.9)
Comorbid		
Yes	24	(12.9)
No	162	(87.1)
eFAST (Extended Focused Assessment with *Sonography* in Trauma)		
Positive	32	(17.2)
Negative	154	(82.8)
Repeat eFAST		
Positive	83	(44.6)
Negative	103	(55.4)
Emergency operative intervention		
Yes	49	(26.3)
No	137	(73.7)
Outcome		
Discharged	178	(95.7)
Died	8	(4.3)
Massive Transfusion		
Yes	49	(26.3)
No	137	(73.7)

The overall mean ± SD was evaluated for quantitative variables i.e., age, weight, GCS, pulse at presentation, systolic BP at presentation, diastolic BP at presentation, MAP at presentation, RR and shock index (SI) at presentation, injury severity score, length of stay, and the number of crystalloids within the first 4 hours of presentation. We observed 102 (55%) patients with low PP. The results are presented in Table 2.

**Table 2:** Descriptive statistics of quantitative variables

	mean±SD	Minimum	Maximum
Age (years)	28.66±11.86	18.0	60.0
Weight (kg)	60.46±12.38	40.0	89.0
GCS (Glasgow coma scale)	14.94±0.23	14.0	15.0
Pulse at presentation	85.47±10.87 beats/min	68.0 beats/min	132.0 beats/min
Systolic Blood Pressure at presentation	110.13±12.2 mmHg	82.0 mmHg	140.0 mmHg
Diastolic Blood Pressure at presentation	79.2±10.13 mmHg	54.0 mmHg	105.0 mmHg
MAP (Mean Arterial Pressure) at presentation	89.41±9.84 mmHg	68.0 mmHg	113.0 mmHg
RR (Respiratory rate) at presentation	20.7±3.43 breaths/min	16.0 breaths/min	34.0 breaths/min
Injury Severity Score (ISS)	16.54±8.63	3.0	38.0
Shock Index (SI)	0.78±0.15	0.5	1.3
Length of stay (hours)	45.69±45.5	4.0	192.0
No. of crystalloids (Liters) within first 4 hours of presentation	2.81±0.9	1.0	5.0
Pulse Pressure at presentation	31.17±9.72 mmHg	16.0 mmHg	55.0 mmHg
No. of transfusions	3.57±4.59	0.0	16.0

According to Table 3, there was a statistically significant association between low PP and gender (p=0.036), length of stay (p=0.000), repeat eFAST (p=0.000), emergency operational intervention (p=0.000), outcome (p=0.009), and MT (p=0.000). Table 4 shows that among patients with low or high PP, the significant mean difference in the number of crystalloids (lt.) consumed within the first four hours after the presentation (p=0.000), injury severity score (p=0.000), SBP (p=0.000), and pulse rate (p=0.002) were significantly associated with low PP.

**Table 3:** Association of low pulse pressure with patient’s characteristics Chi-square/fisher exact test was applied. P-value<0.05 was considered significant. * Significant at 0.01 levels.

	Low Pulse Pressure n (%)		P-Value
	Yes (n=102)	No (n=84)	
Gender			
Male	95 (93.1)	70 (83.3)	0.036
Female	7 (6.9)	14 (16.7)	
Age Group			
≤35 years	85 (83.3)	59 (70.2)	0.089
36-50 years	11 (10.8)	14 (16.7)	
>50 years	6 (5.9)	11 (13.1)	
Length of Stay			
≤24 hours	40 (39.2)	60 (71.4)	0.000*
25-72 hours	24 (23.5)	19 (22.6)	
>72 hours	38 (37.3)	5 (6)	
Mechanism of injury			
Assault	10 (9.8)	12 (14.3)	0.441
Fall	31 (30.4)	29 (34.5)	
Road Traffic Accident	61 (59.8)	43 (51.2)	
Comorbid			
Yes	12 (11.8)	12 (14.3)	0.610
No	90 (88.2)	72 (85.7)	
eFAST (Extended Focused Assessment with *Sonography* in Trauma)			
Positive	21 (20.6)	11 (13.1)	0.178
Negative	81 (79.4)	73 (86.9)	
Repeat eFAST			
Positive	58 (56.9)	25 (29.8)	0.000*
Negative	44 (43.1)	59 (70.2)	
Emergency operative intervention			
Yes	47 (46.1)	2 (2.4)	0.000*
No	55 (53.9)	92 (97.6)	
Outcome			
Discharged	94 (92.2)	84 (100)	0.009*
Died	8 (7.8)	0 (0)	
Massive Transfusion			
Yes	44 (43.1)	5 (6)	0.000*
No	58 (56.9)	79 (94)	

**Table 4:** Mean comparison of patient’s characteristics according to low pulse pressure An Independent t-test was applied. P-value<0.05 was considered significant. * Significant at 0.01 levels.

	Low Pulse Pressure		P-Value
	Yes (n=102)	No (n=84)	
	Mean±SD	Mean±SD	
Age (years)	27.57±10.85	10.85±29.97	0.178
Weight (kg)	58.91±11.91	11.91±62.35	0.059
Glasgow Coma Scale (GCS)	14.911±0.28	0.28±14.97	0.051
Pulse at presentation (beats/min)	87.72±11.63	11.63±82.75	0.002*
Systolic Blood Pressure at presentation (mmHg)	104.94±10.16	10.16±116.44	0.000*
Diastolic Blood Pressure at presentation (mmHg)	80.45±10.28	10.28±77.69	0.064
Mean Arterial Pressure (MAP) at presentation (mmHg)	88.54±10.02	10.02±90.46	0.187
Respiratory Rate (RR) at presentation (breaths/min)	20.85±4.06	4.06±20.52	0.517
Injury Severity Score (ISS)	18.43±8.98	8.98±14.26	0.001*
Shock Index (SI)	0.84±0.14	0.14±0.72	0.000*
Length of stay (hours)	62.07±50.81	50.81±25.8	0.000*
No. of crystalloids (Liters) within first 4 hours of presentation	3.25±0.75	0.75±2.27	0.000*

## Discussion

The present study was conducted to evaluate the utilization of low PP as an independent predictor of MT or operative intervention. In our study, males were more frequent victims of RTA than women. Amongst 85.8% of RTA victims with negative eFAST, only 26.3% underwent emergency operative intervention with mortality outcomes of 4.3% (eight patients). The need for MT was observed in 26.3% of them and 55% (102) had low PP. A statistically significant association was observed between low PP and gender, length of stay, repeat eFAST, emergency operational intervention, outcome, MT, number of crystalloids (Lt.) consumed within the first four hours after presentation, injury severity score, SBP, and pulse rate.

Bleeding is the number one cause of preventable death in trauma patients, and all efforts should be directed at detecting the source of bleeding, stopping the bleeding, and replacing lost volume [1]. Aside from individual vital signs, scoring systems have also been developed to identify life-threatening hemorrhages. Several of these have been tested, but have sensitivities of only 50% to 60% [22]. The pulse rate over pressure evaluation index, which is calculated by heart rate over PP, was found to have a sensitivity of 55%, with a specificity of 79%, in its ability to identify those who will decompensate in the ED [23].

PP is defined as the difference between systolic and diastolic blood pressure. Significant blood loss, as in the trauma population, can yield decreases in PP [24,25]. One major advantage of using PP compared to other scoring systems is that it requires limited data without the need for calculations and does not rely upon laboratory values, which may result in unnecessary delays. Narrowed PP may provide a new measure to help triage patients’ level of care upon their admission with a simple blood pressure measurement. In prior studies, the PP and heart rate (PP/HR) ratio indicates the need for MT [17,26].

Certain populations may have elevated PP that exists at the baseline. Young, athletic patients have progressively increased stroke volume, cardiac output during exercise, and decreased total peripheral resistance; this combination yields a higher PP. The geriatric population may also have a higher PP, but secondary to decreased compliance of large arteries or left ventricle. These changes may yield cardiac contractility against a stiffer arterial system, worsening subsequent hypertrophy, and widening PP further. A cutoff analysis in the study by Priestly et al. showed an inflection at 55 mmHg for patients ≥61 years old, and 40 mmHg for adult patients ≤60 years old, and this showed no statistical differences among varying ages in a study by Warren et al. [4].

Zhu et al. assessed a smaller cohort of adult patients requiring MT, alternatively found their population with narrowed PP to more often have blunt injuries, and that narrowed PP was again a positive predictor for mortality [27]. Finally, a recent study by Priestly et al. of 18,000 trauma patients examined retrospectively showed that a narrow initial ED PP was an independent predictor of active hemorrhage (AH).

In a study by Warner [4], they found that a narrowed PP upon arrival in the ED was associated with the administration of an MT, as well as the need for emergent operative intervention. However, narrowed PP was less specific for those injuries occurring to the face or head. Therefore, although a narrowed PP may not be an ideal screening tool to determine which trauma patients may require an intervention, among patients at risk, the high specificity of this finding suggests that there would be very few false positives and a high probability of the need for an emergent intervention in patients with a positive finding [4,28]. A previous study by Convertino et al. in healthy males demonstrated that PP decreased linearly with the degree of central hypovolemia and that PP is positively correlated with stroke volume (r2=0.9) [29]. Controlled experimental hemorrhage studies have also demonstrated an association between hemorrhage and a decrease in PP [30].

In another study by Priestley [1], PP correlated well with patients requiring transfusion and further intervention for hemorrhage control. Although only 1.6% of the study population met the definition of AH, these patients had a statistically significant narrowing of PP that was greater than that in the non-active hemorrhage (AH) group and was found to have more significant injuries during interventional radiologic (IR) and operative exploration [1]. Furthermore, narrowed ED PP was shown not only to be an independent predictor of AH but as it narrows, the predicted probability of AH increases. PP is a standalone early warning that bleeding is occurring and can easily be obtained and repeated in the resuscitation area with standard monitoring. Although previous studies have discussed its utility in combination with other clinical data, we now know that PP on its own can be a critical early data point in assessing trauma patients, especially in the setting of normal SBP. The presence of narrowed PP should raise suspicion that there is occult, ongoing blood loss, and a search for the source is warranted [1].

The PP response also varies by age. Older patients are unable to physiologically compensate in the same manner as younger patients. As such, and not surprisingly, for patients more than 60 years old, the PP cut-off was 15 mmHg higher than that seen in younger patients. The PP can be quickly calculated in the resuscitation bay regardless of age. Therefore, even in normotensive patients, a search for potential sources is warranted if PP is narrowed due to the strong association with AH [1].

## Conclusions

PP is a simple and rapidly calculated variable that can be used early in the resuscitation period to help guide the treatment of patients injured due to trauma. The cut-off value of PP i.e., PP<30 mmHg was observed as a useful predictor for increased blood loss requiring blood transfusion or operative intervention.
